# Effect of inoculants and storage temperature on the microbial, chemical and mycotoxin composition of corn silage

**DOI:** 10.5713/ajas.17.0801

**Published:** 2018-01-26

**Authors:** Musen Wang, Shengyang Xu, Tianzheng Wang, Tingting Jia, Zhenzhen Xu, Xue Wang, Zhu Yu

**Affiliations:** 1 Department of Grassland Science, China Agricultural University, Beijing 100193, China; 2 Institute of Quality Standards and Testing Technology for Agricultural Products, Chinese Academy of Agricultural Science, Beijing 100081, China

**Keywords:** *Lactobacillus plantarum*, *Pediococcus pentosaceus*, Corn Silage, Nutritive Value, Mycotoxin

## Abstract

**Objective:**

To evaluate the effect of lactic acid bacteria and storage temperature on the microbial, chemical and mycotoxin composition of corn silage.

**Methods:**

Corn was harvested at 32.8% dry matter, and chopped to 1 to 2 cm. The chopped material was subjected to three treatments: i) control (distilled water); ii) 1×10^6^ colony forming units (cfu)/g of *Lactobacillus plantarum*; iii) 1×10^6^ cfu/g of *Pediococcus pentosaceus*. Treatments in triplicate were ensiled for 55 d at 20°C, 28°C, and 37°C in 1-L polythene jars following packing to a density of approximately 800 kg/m^3^ of fresh matter, respectively. At silo opening, microbial populations, fermentation characteristics, nutritive value and mycotoxins of corn silage were determined.

**Results:**

*L. plantarum* significantly increased yeast number, water soluble carbohydrates, nitrate and deoxynivalenol content, and significantly decreased the ammonia N value in corn silage compared with the control (p<0.05). *P. pentosaceus* significantly increased lactic acid bacteria and yeast number and content of deoxynivalenol, nivalenol, T-2 toxin and zearalenone, while decreasing mold population and content of nitrate and 3-acetyl-deoxynivalneol in corn silage when stored at 20°C compared to the control (p<0.05). Storage temperature had a significant effect on deoxynivalenol, nivalenol, ochratoxin A, and zearalenone level in corn silage (p<0.05).

**Conclusion:**

*Lactobacillus plantarum* and *Pediococcus pentosaceus* did not decrease the contents of mycotoxins or nitrate in corn silage stored at three temperatures.

## INTRODUCTION

Corn is widely utilized as a ensiling material throughout the world, and corn plants in the field can be contaminated with fungi and mycotoxin formation may occur under unfavorable conditions, such as high temperature and drought stress. These fungi and mycotoxins produced can survive the ensiling process and cause animal health problems. Mycotoxins are low molecular weight secondary metabolites formed mainly by *Aspergillus*, *Penicillium*, and *Fusarium* species [1]. The most common mycotoxins in livestock feeds are aflatoxin B_1_ (AFB_1_), ochratoxin A (OTA), zearalenone (ZEA) and trichothecenes. Mycotoxin contamination not only results in reduced animal feed intake, reproduction and feed conversion efficacy [2], but also may cause carcinogenesis, teratogenesis and immune system suppression as a result of chronic toxicity even at low levels [3,4]. Moreover, the presence of multiple mycotoxins is of particular concern due to potential synergistic effects on livestock exposed to moldy silage [3]. Another issue is that mycotoxins show higher resistance than mycelia to feedstuff processing and storage [5]. Thus, it is important to identify the methods for degrading or transforming mycotoxins in silage. Physical and chemical approaches, such as an addition of ammonia and adsorbents [4,6], may be dangerous to applicators. In addition, some adsorbents even bind minerals and vitamins as well as mycotoxins, reducing feed quality [7].

A few studies have indicated that mycotoxins can be degraded or transformed by some microbes. Rumen microflora can degrade and inactivate mycotoxins [8], and intestinal microbes were shown to convert ZEA to α-zearalenol and an unknown metabolite [9]. Some reduction of mycotoxins produced in the field was attributed to lactic acid bacteria (LAB) [10]; an *in vitro* study reported that binding of (deoxynivalenol) DON and ZEA is the major mode of action for LAB [11]. Microbial activity during silage fermentation caused the breakdown of mycotoxin ZEA [12], and *Lactobacilli* and *Pediococcus* species were reported to be able to transform some mycotoxins [13]. Mold normally grows at 10°C to 40°C, and *Aspergillus* and *Penicillium* species can grow at higher temperatures than *Fusarium* species [14]. However, mold growth under the optimum temperature range is not necessary for mycotoxins production. A comparison of the optimum temperature for mycotoxins formation by *Aspergillus*, *Penicillium* and *Fusarium* species [15], defined the temperature range of mycotoxins production for *A. flavus* (AFB_1_), *P. verrucosum* (OTA) and *Fusarium* species (toxin T-2, DON, nivalenol [NIV] and ZEA) to be 12°C to 40°C, 4°C to 20°C, and 24°C to 26°C, respectively. To date, few studies have evaluated the effect of LAB on the stability of mycotoxins in corn silage stored at different temperatures.

Thus, the objective of this study was to evaluate the effect of inoculants and storage temperature on the microbial populations, fermentation characteristics, nutritive value and mycotoxins of corn silage.

## MATERIALS AND METHODS

### Silage preparation

Corn was grown at the Zhuozhou experimental farm of China Agricultural University (Hebei Province: N 39°35′25″-39°36′05″, E 115°42′12″-116°14′35′, elevation 22 to 65 m, annual mean temperature 11.6°C, and annual average precipitation 554 mm). Organic matter, hydrolyzable N, available P and rapidly available K content in the field was 16,000, 89, 26.2, and 83 mg/kg, respectively. The cultivar ‘Xianyu045’ was established on 8 June 2016, with about 64,000 plants/hectare. A compound fertilizer (N+P_2_O_5_+K_2_O ≥45%, 800 kg/hectare) was applied preplant, and urea (320 kg/hectare) was supplemented at the fourteenth-leaf stage on 18th July 2016. A 3×3 factorial arrangement was used in a completely randomized design. Corn was harvested at 32.8% dry matter (DM), and chopped to 1 to 2 cm with a forage chopper on 8th September 2016. The chopped forage was subjected to three treatments: i) control (distilled water, CK); ii) 1×10^6^ colony forming units (cfu)/g of *Lactobacillus plantarum* KR107070 (LP), isolated from Chinese wild rye (*Leymus chinensis*) [16]; iii) 1×10^6^ cfu/g of *Pediococcus pentosaceus* 17604 (PP), isolated from alfalfa silage. Treated forage in triplicate was ensiled in 1-L polythene jars (Hewanglan Paper and Plastic Products Factory, Beijing, China) following packing with a cylindrical rod and an axe to a density of about 800 kg of fresh weight/m^3^. Laboratory silos were stored at 20°C, 28°C, and 37°C for 55 d, respectively, with 27 silos in all.

### Chemical and microbial analyses

At silo opening, corn silage mass was mixed manually prior to sampling. A subsample of 20 g was weighed into a blender jar, diluted with 180 mL of distilled water, and homogenized with a juicer for 2 min. Extracted solution was filtered through four layers of cheesecloth and one layer of qualitative filter paper, and analyzed for pH value using an electrode (PHS-3C, INESA, Shanghai, China). Then, 2 mL of filtrate was centrifuged at 10,000 g at 4°C for 5 min and reserved for fermentation acids and ammonia N (NH_3_-N) analysis. Lactic, acetic, propionic, and butyric acids were determined by high performance liquid chromatograph (HPLC, SHIMADZU-10A, Kyoto, Japan). The HPLC system consisted of a Shimadzu system controller (SCL-10A), and a Shodex Rspak KC-811 S-DVB gel column (300 mm×8 mm) with a column temperature of 50°C. The mobile phase was 3 mmol HClO_4_ running at 1 mL/min, and the injection volume was 5 μL. A UV detector (SPD-10A) was used for detection at 210 nm. NH_3_-N was determined by the phenol method.

A second subsample of 200 g from the ensiled forage or silage was dried in a forced-draft oven at 65°C for at least 48 h to determine DM. Neutral detergent fiber and acid detergent fiber were determined according to Van Soest et al [17]. Water soluble carbohydrates (WSC) were determined using the anthrone method. Nitrogen was analyzed according to Kjeldahl method. Crude protein was calculated by multiplying 6.25 with N content. Ether extract (EE) was determined by petroleum ether extraction using filter bag technology. A XT4 filter bag was filled with 1 g of sample, sealed with a capper, and extracted in a X15I FAT EXTRACTOR (ANKOM Technology Corp., Macedon, NY, USA) with petroleum ether (analytical reagent grade, 30°C to 60°C) for 1 h. Petroleum ether was purchased from Sinopharm Chemical Reagent Co., Ltd (Beijing, China). Nitrate was determined by the salicylic acid method.

The third subsample of 20 g from silage was put into a sterile triangular flask, suspended in 180 mL of sterile solution (1 g of peptone and 9 g of sodium chloride per liter), and homogenized in a laboratory oscillator at a low speed for 30 min. A series of dilute solutions were prepared for microbial count. The LAB were measured on MRS agar by incubating plates at 37°C for 2 d under anaerobic conditions. Coliform bacteria (CB) were estimated following growth on violet red bile agar incubated at 37°C for 3 d. Yeast and mold were determined on rose bengal medium after incubation at 28°C for 3 d and 5 to 7 d, respectively, with yeast and mold numbers counted separately according to their macromorphological characteristics. Bacteria and fungi were counted on the plates that yield 30 to 400 cfu and 1 to 100 cfu, respectively. All media used were obtained from Beijing Aoboxing Biotech Co., Ltd (Beijing, China).

### Mycotoxins determination

#### Reagents and solutions

Mycotoxin standard solution NIV (100 μg/mL), DON (100 μg/mL), 3-acetyl-deoxynivalneol (3-ADON, 100 μg/mL), AFB_1_ (2 μg/mL), HT-2 (100 μg/mL), T-2 (100 μg/mL), OTA (10 μg/mL), and ZEA (10 μg/mL) was obtained from Romer Lab (Romer Labs Inc., Newark, NJ, USA). Diluted solutions were prepared immediately before use by diluting the standard solutions with methanol. A stock solution was composed of eight mycotoxins described above, and prepared by combining suitable aliquots of each individual standard dilution to make each mycotoxin 1 μg/mL. A working solution of mycotoxin mixtures was made using a stock solution of the following concentrations: 0.5, 1, 5, 10, 20, 50, and 100 ng/mL. Liquid chromatograph-mass spectrometry grade methanol was purchased from J&K Scientific Ltd (Beijing, China). Acetic acid (guaranteed reagent grade) was obtained from Sigma-Aldrich (Shanghai, China). All reagents (analytical reagent grade) were supplied by Yanxiwan Chemical Reagent Company (Beijing, China), and 15 and 50 mL polyethylene centrifuge tubes used were purchased from Corning Inc. (Corning, NY, USA).

#### Extraction procedure and clean-up

For silage extracting, the QuEChERS extraction method was used [18,19], where 5 g of DM silage was weighed into a 50 mL centrifuge tube, soaked in 10 mL of 2% formic acid for 30 min, and oscillated on a rotary shaker for 30 min at 240 rpm after an addition of 10 mL of acetonitrile. Subsequently, 4 g of MgSO_4_ and 1 g of NaCl were added, and the tube was capped immediately (a brief hand shaking was done immediately after an addition of salts to prevent agglomeration). The slurry was immediately oscillated for 30 s with a vortex mixer and centrifuged at 10,000 g for 5 min at 4°C, with an aim to induce phase separation and mycotoxins partitioning. Removal of residual water and cleanup were performed simultaneously by using a rapid procedure called dispersive solid-phase extraction (dispersive-SPE), where 2 mL of acetonitrile extract was mixed with 300 mg of MgSO_4_ and 100 mg of C_18_ endcapped silica sorbent (Agilent Technologies, Santa Clara, CA, USA) in a 15 mL centrifuge tube, and centrifuged at 10,000 g for 1 min at 4°C. A portion of the final solution (1 mL) was filtered to a sample bottle (Agilent Technologies, USA) by a 0.22 μm syringe filter, and stored at −20°C until further analysis.

#### Instrumental conditions

**Figure 1:**
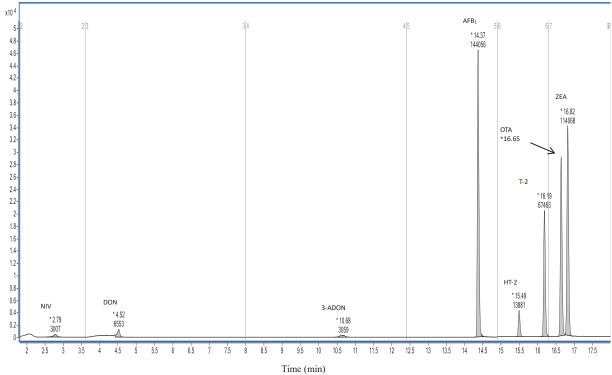
The chromatogram of nivalenol, deoxynivalenol, 3-acetyl-deoxynivalneol, aflatoxin B_1_, HT-2 toxin, T-2 toxin, ochratoxin A, and zearalenone at 100 ng/mL.

Instrumental conditions were set according to the Agilent Mass Spectrometry (MS) Technology Products Solutions solution package (mycotoxin, 1290-6470 Parameters, Agilent Technologies, USA). An Agilent infinity 1290 ultra-high performance liquid chromatography (UHPLC) system (Agilent Technologies, USA) was utilized throughout the study. This instrument consisted of a UHPLC pump with a built-in micro-degasser, an infinity autosampler with back-flush function, and a temperature control compartment. Chromatographic separation was achieved using an Agilent Eclipse Plus column (1.8 μm, 100 mm×2.1 mm, Agilent Technologies, USA) at a flow rate of 300 μL/min, with a mobile phase consisting of solvent A and solvent B, where solvent A was water solution containing 1% acetic acid and 5 mM ammonium acetate, and solvent B was methanol. The chromatogram of 8 mycotoxins mixed standard solution and gradient elution program are shown in Figure 1 and Table 1, respectively. The injection volume of each sample was 2 μL and column temperature was 40°C.

Analytes were detected using an Agilent 6470 triple quadrupole MS equipped with a Jet Stream electrospray ionization probe. MS was operated in dynamic multi-reaction monitor by monitoring two transitions (one quantifier and the other qualifier) for each analyte, with individual dwell time. MS parameters were as follows: gas temperature 300°C; sheath gas temperature 350°C; gas flow 7 L/min, sheath gas flow 11 L/min, and capillary 3,500 V, positive. The acquisition was performed in positive polarity, and the optimized MS conditions are outlined in Table 2. Linearity was established by injecting increasing concentrations (triplicates) of working solution (0.5, 1, 5, 10, 20, 50, and 100 ng/mL). Standard curves were linear in the range studied, showing correlation coefficients of >0.999. Quantification and detection limits were determined by spiked samples based on signal-to-noise ratios of 10:1 for quantification and 3:1 for detection limit.

### Statistical analysis

All microbial counts (LAB, yeast, mold, and CB) were transformed to log10 units on fresh weight basis. Data of the fermentation characteristics, microbial populations, nutritive value and mycotoxin content in silage were analyzed by the Factorial Design model of SAS (9.1 version, SAS Institute Cary, NC, USA). The model used for the analysis was: *Y* = *μ* + treatment + storage temperature + treatment × storage temperature + *ɛ*, where *Y* = observation, *μ* = general mean, treatment = effect of CK or LP or PP, storage temperature = effect of 20°C or 28°C or 37°C, treatment×storage temperature = interaction between treatment and storage temperature, *ɛ* = residual error. Bon grouping contrasts were utilized to compare means among four levels of one factor with the other factor immobilized. The significant difference was declared at p<0.05.

## RESULTS

### Chemical, microbial and mycotoxin composition of corn

Chemical and microbial composition and mycotoxin content of ensiling plant are presented in Table 3 and 4, respectively. Nitrate, DON, and ZEA content of corn was 383 mg/kg, 163 and 30.6 ng/g accordingly.

### Microbial and chemical composition of corn silage

Table 5 shows the microbial counts and fermentation characteristics of corn silage treated with inoculants and storage temperature. LAB count in PP-treated silage silage stored at 20°C was higher than CK and LP (p<0.05), and their count in PP-treated silage silage stored at 28°C was higher than LP (p<0.05), whereas LP-treated silage stored at 37°C had a higher count of LAB compared with CK and PP (p<0.05). Without any treatment, LAB number in silage conserved at 28°C was higher in comparison with 20°C and 37°C (p<0.05), and their number in silage conserved at 20°C was higher compared with 37°C (p<0.05). The addition of PP resulted in a lower population of LAB in silage conserved at 37°C in contrast with 20°C and 28°C (p<0.05). Yeast number in any silage decreased as storage temperature increased from 20°C to 37°C (p<0.05), regardless of additive effect. Yeast number in LP-treated silage ensiled at any temperature was higher than CK (p<0.05), and its number in PP-treated silage ensiled at 20°C and 28°C was higher than CK (p<0.05), whereas PP-treated silage ensiled at 20°C had a higher count of yeast in comparison with LP (p<0.05). With the addition of PP or no additive, yeast population in silage ensiled at 20°C was higher compared to 28°C and 37°C (p<0.05). Mold count in PP-treated silage stored at 20°C was lower than CK and LP (p<0.05). Without any additive, silage stored at 20°C had a higher count of mold in contrast with 37°C (p<0.05), whereas the application of PP led to a higher number of mold in silage stored at 28°C compared with 20°C and 37°C (p<0.05). CB number in CK-treated silage stored at 37°C was lower compared to LP and PP (p< 0.05), and CK-treated silage stored at 20°C had a higher number of CB in comparison with 28°C and 37°C (p<0.05).

According to Table 5, the pH value in any silage was below 4.0, ranging from 3.69 to 3.85. The pH value in PP-treated silage conserved at 20°C and 28°C was higher than CK and LP (p<0.05). With the addition of LP or no additive, silage conserved at 37°C had a higher pH level compared to 20°C and 28°C (p<0.05). The application of PP not only resulted in a higher pH level in silage conserved at 37°C in comparison with 20°C and 28°C (p<0.05), but also caused a higher value of pH in silage conserved at 20°C compared to 28°C (p<0.05). NH_3_-N content in any silage increased significantly as conservation temperature increased from 20°C to 37°C (p<0.05), irrespective of treatment effect. NH_3_-N content in LP-treated silage stored at any temperature was lower than CK (p<0.05), whereas PP-treated silage stored at 28°C had a lower level of NH_3_-N in comparison with CK (p<0.05). The addition of LP lowered NH_3_-N level in silage stored at 20°C and 37°C compared to PP (p<0.05). Without any additive, NH_3_-N value in silage ensiled at 28°C and 37°C was higher compared to 20°C (p<0.05). With the use of LP, NH_3_-N value in silage ensiled at 37°C was higher compared to 20°C and 28°C (p<0.05), and its content in silage ensiled at 28°C was higher than 20°C (p<0.05). The application of PP led to a higher content of NH_3_-N in silage ensiled at 37°C compared with 20°C and 28°C (p<0.05). PA content in LP-treated silage ensiled at 20°C was higher than 28°C and 37°C (p<0.05), whereas there were not significant differences on PA content for all the other contrasts (p>0.05).

Table 6 presents the effect of inoculants on the nutritive value of corn silage stored at three temperatures. DM content in CK-treated silage stored at 37°C was lower in comparison with LP and PP (p<0.05), and there were not significant differences on DM content for all the other contrasts (p>0.05). CP level in any silage decreased significantly with storage temperature increasing from 20°C to 37°C (p<0.05), irrespective of additive effect. CP level in CK-treated silage conserved at 28°C was lower compared to LP and PP (p<0.05), whereas its content in LP-treated silage conserved at 37°C was higher than CK and PP (p<0.05). With no additive, silage conserved at 20°C had a higher content of CP compared with 28°C and 37°C (p<0.05). The application of LP induced a higher value of CP in silage conserved at 20°C in contrast with 37°C (p< 0.05), and PP-treated silage conserved at 37°C had a lower value of CP than 20 and 28°C (p<0.05). WSC content in LP-treated silage ensiled at any temperature was higher compared to CK and PP (p<0.05), whereas its content in PP-treated silage ensiled at 37°C was higher than CK (p<0.05). With the addition of PP or no additive, silage ensiled at 37°C had a higher level of WSC in comparison with 20°C and 28°C (p<0.05), and WSC level in silage ensiled at 20°C was higher in contrast with 28°C (p<0.05). The application of LP resulted in a higher value of WSC in silage ensiled at 37°C compared to 20°C and 28°C (p<0.05), whereas there were no differences on WSC value for all the remaining comparisons (p>0.05). Nitrate content in silage, ranging from 61.1 to 91.4 mg/kg, was lower compared with 383 mg/kg in corn. At 20°C, nitrate level in LP-treated silage was higher compared to CK and PP (p<0.05), and its level in silage treated with CK was higher than PP (p<0.05). LP-treated silage stored at 28°C had a higher level of nitrate than CK and PP (p<0.05). At 37°C, nitrate level in silage treated with LP was higher compared to CK and PP (p<0.05), and its level in silage treated with CK was lower in comparison with PP (p<0.05). The use of LP led to a higher value of nitrate in silage stored at 20°C compared to 28°C (p<0.05), whereas PP-treated silage stored at 37°C had a higher value of nitrate than 20°C and 28°C (p<0.05), and the application of PP caused a lower value of nitrate in silage stored at 20°C in contrast with 28°C (p<0.05).

### Mycotoxin content of corn silage

Mycotoxin content of corn silage treated with LAB and storage temperature is stated in Table 7. NIV content in PP-treated silage stored at 20°C was higher than CK and LP (p<0.05), whereas its content in PP-treated silage stored at 28°C and 37°C was lower compared to CK and LP (p<0.05). NIV level in LP-treated silage was higher and lower in comparison with CK (p<0.05), at 28°C and 37°C, respectively. Without any additive, NIV value in silage stored at 37°C was higher compared to 20°C and 28°C (p<0.05), and its value in silage stored at 20°C was lower than 28°C (p<0.05). The use of LP resulted in a lower value of NIV in silage stored at 20°C in contrast with 28°C and 37°C (p<0.05). NIV level in PP-treated silage stored at 37°C was higher compared with 20°C and 28°C (p< 0.05), and its level in silage stored at 20°C was higher than 28°C (p<0.05). DON content in PP-treated silage conserved at 20°C was higher compared to CK and LP (p<0.05), and its content in LP-treated silage conserved at 28°C was higher in comparison with CK and PP (p<0.05). At 37°C, DON level in silage treated with LP was higher than CK and PP (p<0.05), and its level in silage treated with CK was lower compared with PP (p<0.05). With no additive, silage conserved at 20°C had a higher value of DON in contrast with 28°C and 37°C (p<0.05). DON value in silage treated with LP increased significantly from 76.2 to 179 ng/g as storage temperature increased from 20°C to 37°C. With the application of PP, DON level in silage ensiled at 20°C was much higher compared to 28°C and 37°C (p<0.05), and its level in silage ensiled at 28°C was lower than 37°C (p<0.05). At 20°C, the 3-ADON level in silage treated with CK was higher in comparison with LP and PP (p<0.05), whereas there were no differences on 3-ADON level for all the remaining comparisons (p>0.05). PP-treated silage stored at 20°C had a higher level of T-2 compared to CK and LP (p<0.05). OTA value in PP-treated silage ensiled at 28°C was higher compared with CK and LP (p<0.05). When storage temperature increased to 37°C, silage treated with CK had a higher content of OTA in contrast with LP and PP (p<0.05), and LP-treated silage had a lower content of OTA than PP (p<0.05). Without any additive, OTA level in silage ensiled at 37°C was higher compared to 20°C and 28°C (p<0.05). OTA level in PP-treated silage ensiled at 37°C was higher in comparison with 20°C and 28°C (p<0.05), and its level in PP-treated silage ensiled at 20°C was lower than 28°C (p<0.05). ZEA content in PP-treated silage conserved at 20°C and 28°C was higher compared with CK and LP (p<0.05). The utilization of LP led to a higher value of ZEA in silage conserved at 37°C in comparison with 28°C (p<0.05), whereas ZEA value in PP-treated silage conserved at 28°C was higher than 20°C and 37°C (p<0.05).

## DISCUSSION

In the present experiment (Table 5), LP-treated corn silage stored at any temperature had a lower level of NH3-N and a higher number of yeast in comparison with CK, which is in agreement with Filya et al [20]. Without any additive, LAB number in corn silage stored at 37°C was lower than 20°C and 28°C, and a study found the similar phenomenon [21]. According to Table 6, the application of LP resulted in a higher content of WSC in corn silage stored at any temperature compared to CK, it was reported that its concentration in corn silage treated with LP was lower than CK [20]. EE content decreased from 2.69% in corn to 2.09% to 2.29% of corn silage, perhaps microbial activity during fermentation degraded some EE. Nitrate level in corn was 383 mg/kg, and decreased to 61.1 to 91.4 mg/kg after ensiling. Both corn and silage were safe in terms of nitrate content, because their concentrations were below the limit level (1,000 mg/kg). A study showed that nitrate contents in corn were 374 and 433 mg/kg, and reduced to 18.5 to 111 mg/kg of corn silages [22].

The eight mycotoxins in this experiment can be divided into *Aspergillus* and *Fusarium* toxins, with AFB_1_ and OTA belonging to *Aspergillus* toxins, and *Fusarium* toxins consisting of the remaining six toxins. As can be seen from Table 4 and 7, AFB_1_ existed in pre- and post-fermented corn samples, and its content was higher in corn (30.2 ng/g) compared with corn silage (23.4 to 24.5 ng/g). This result indicated that AFB_1_ had a slow degradation during fermentation, and agrees with Kalac and Woodford [23]. However, AFB_1_ level was higher in corn silage when compared with pre-fermented samples [24], suggesting that *A. flavus* and *A. parasiticus* activity was enhanced during storage. Silage contamination with AFB_1_ was also found in Argentina [25], Egypt [26] and France [27]. These phenomena above show that AFB_1_ infection may be influenced by field environment where forage crop grow, and is usually concerned with tropical or subtropical regions with a higher temperature. Treatment and storage temperature did not have an effect on AFB_1_ level in corn silage at this experiment, however. OTA is a common contaminant of corn in temperate regions, and its level in corn was 34.4 ng/g at this experiment. Conservation temperature has a significant effect on OTA content in corn silage, and the application of LP and PP decreased its level in corn silage stored at 37°C compared to CK.

Most information for *Fusarium* toxins is for DON in corn silage. DON content in CK-treated corn silage stored at any temperature was lower than that of corn (Tables 4, 7), and two workers found a significant decrease in DON content after ensiling [28], perhaps because *Fusarium* species belong to field molds and their activity was suppressed by low oxygen and pH environment of silage [29]. In this experiment, storage temperature had a remarkable effect on DON level, and its level was not reduced in corn silage treated with LP and PP, whereas one study demonstrated that storage temperature did not affect DON stability [28]. Furthermore, their results showed that ensiling with low DM and prolonging storage time led to mycotoxin degradation of corn silage. DON level was also influenced by silage distribution in a silo, proved by two studies. One study showed that a higher content of DON was detected on the top of corn silage [27], and a lower concentration of it was found at the bottom of corn silage by the same workers [27]. On the whole, ZEA level kept unchanged in corn silage in this study, and this result was in accordance with Gonzales Pereyra et al [25].

## Figures and Tables

**Table 1:** Elution condition for the mobile phase

Time (min)	Solvent A (%)	Solvent B (%)
0	90	10
2	90	10
3	80	20
7	76	24
10.5	70	30
13.5	40	60
15	30	70
18	25	75
18.1	5	95
21.9	5	95
22	90	10
25	90	10

**Table 2:** Chromatographic and mass spectrometric parameters for the detection of mycotoxins

Analyte	Precursor ion	Product ion (quantification)	Product ion (qualifier)	Dwell time (ms)	Collision energy (V)	Retention time (min)	Limit of detection (ng/g)	Limit of quantification (ng/g)
NIV	371	281	59.1	100	10	2.79	1	5
DON	355	265	59	60	10	4.52	1	5
3-ADON	339	231	213	100	10	10.6	1	5
AFB_1_	313	285	241	30	22	14.3	0.5	1
HT-2	442	263	215	40	10	15.4	1	5
T-2	484	305	185	40	10	16.1	0.3	1
OTA	404	239	358	20	20	16.6	0.5	1
ZEA	317	175	130	20	25	16.8	0.5	1

NIV, nivalenol; DON, deoxynivalenol; 3-ADON, 3-acetyl-deoxynivalneol; AFB_1_, aflatoxin B_1_; HT-2, HT-2 toxin; T-2, T-2 toxin; OTA, ochratoxin A; ZEA, zearalenone.

**Table 3:** Chemical and microbial composition of corn material

Item	DM	NDF	ADF	CP	WSC	EE	Nitrate	LAB	Yeast	Mold	CB

	----------------------------------------------- % -------------------------------------------						mg/kg, DM	--------------------- Log10 cfu/g, FM ---------------			
Mean±SD	32.81±1.71	45.19±1.80	22.85±1.28	8.29±0.36	6.75±0.43	2.69±0.11	383.6±8.72	4.17±0.05	5.99±0.89	5.18±1.12	7.35±1.28

DM, dry matter; NDF, neutral detergent fiber; ADF, acid detergent fiber; CP, crude protein; WSC, water soluble carbohydrates; EE, ether extract; LAB, lactic acid bacteria; CB, coliform bacteria; FM, fresh matter; SD, standard deviation.

Data were shown with triplicate.

**Table 4:** Mycotoxin content of corn material

Item	NIV	DON	3-ADON	AFB_1_	HT-2	T-2	OTA	ZEA

	----------------------------------------------------------------------- ng/g, dry matter -------------------------------------------------------------------							
Mean±SD	37.53±2.86	163.2±11.18	36.98±0.47	30.26±0.77	13.94±0.82	23.01±0.14	34.45±0.27	30.63±2.64

NIV, nivalenol; DON, deoxynivalenol; 3-ADON, 3-acetyl-deoxynivalneol; AFB_1_, aflatoxin B_1_; HT-2, HT-2 toxin; T-2, T-2 toxin; OTA, ochratoxin A; ZEA, zearalenone; SD, standard deviation.

Data were shown with triplicate.

**Table 5:** Microbial populations and fermentation characteristics of corn silage treated with inoculants and storage temperature

Item	Treatment	Storage temperature (°C)			p-value		
	
		20	28	37	T	S	T×S
LAB	CK	6.51^bB^	7.37^abA^	5.33^bC^	0.0148	<0.0001	0.0023
	LP	6.26^b^	6.43^b^	5.90^a^	-	-	-
	PP	7.33^aA^	7.79^aA^	5.26^bB^	-	-	-
Yeast	CK	5.35^cA^	3.83^bB^	3.55^bB^	<0.0001	<0.0001	0.3244
	LP	6.03^b^	5.52^a^	5.00^a^	-	-	-
	PP	6.34^aA^	4.88^aB^	4.87^abB^	-	-	-
Mold	CK	4.01^aA^	3.47^AB^	2.80^B^	0.5366	0.0097	0.0424
	LP	3.38^ab^	3.36	2.80	-	-	-
	PP	2.80^bB^	3.99^A^	3.00^B^	-	-	-
CB	CK	5.74^A^	4.42^B^	4.08^bB^	0.2889	0.0946	0.1605
	LP	5.13	5.34	5.03^ab^	-	-	-
	PP	5.30	5.07	5.07^a^	-	-	-
pH	CK	3.71^bB^	3.71^bB^	3.81^A^	0.0005	<0.0001	0.2219
	LP	3.69^bB^	3.70^bB^	3.83^A^	-	-	-
	PP	3.80^aB^	3.75^aC^	3.85^A^	-	-	-
NH_3_-N	CK	2.98^aB^	3.98^aA^	4.38^aA^	<0.0001	<0.0001	0.2937
	LP	1.90^bC^	2.78^bB^	3.50^bA^	-	-	-
	PP	3.01^aB^	3.34^bB^	4.26^aA^	-	-	-
LA	CK	4.91	5.16	5.23	0.1725	0.3900	0.6227
	LP	5.53	5.76	5.54	-	-	-
	PP	5.19	5.57	5.56	-	-	-
AA	CK	1.45	1.42	1.56	0.1202	0.4850	0.7580
	LP	1.53	1.41	1.40	-	-	-
	PP	1.39	1.26	1.30	-	-	-
PA	CK	0.31	0.31	0.28	0.4567	0.0027	0.2637
	LP	0.39^A^	0.30^B^	0.28^B^	-	-	-
	PP	0.35	0.30	0.28	-	-	-

T, treatment effect; S, storage temperature effect; T×S, interaction effect between treatment and storage temperature; LAB, lactic acid bacteria; CK, control; LP, *Lactobacillus plantarum* KR107070; PP, *Pediococcus pentosaceus* 17604; CB, coliform bacteria; NH_3_-N, ammonia nitrogen; LA, lactic acid; AA, acetic acid; PA, propionic acid.

Fermentation indexes are expressed on dry matter (%) except pH and NH_3_-N, and microbial numbers are shown on a log_10_ unit basis (fresh matter). NH_3_-N is shown on total nitrogen basis (%).

Means within columns (^a,b,c,d^) or rows (^A,B,C,D^) with different letters are significantly different (p<0.05).

**Table 6:** Nutritive value of corn silage treated with inoculants and storage temperature

Item	Treatment	Storage temperature (°C)			p-value		
	
		20	28	37	T	S	T×S
DM	CK	33.4	33.5	32.4^b^	0.0318	0.7671	0.3377
	LP	32.9	33.3	34.2^a^	-	-	-
	PP	34.2	34.7	34.3^a^	-	-	-
CP	CK	7.53^A^	7.04^bB^	6.99^bB^	0.0007	0.0002	0.2021
	LP	7.75^A^	7.57^aAB^	7.36^aB^	-	-	-
	PP	7.52^A^	7.52^aA^	7.19^bB^	-	-	-
WSC	CK	0.98^bB^	0.83^bC^	1.19^cA^	<0.0001	<0.0001	0.0005
	LP	1.31^aB^	1.41^aB^	1.65^aA^	-	-	-
	PP	0.98^bB^	0.78^bC^	1.43^bA^	-	-	-
EE	CK	2.12	2.26	2.22	0.6179	0.6213	0.7625
	LP	2.17	2.10	2.11	-	-	-
	PP	2.09	2.29	2.26	-	-	-
Nitrate	CK	73.9^b^	71.4^b^	67.6^c^	<0.0001	0.2883	0.0002
	LP	91.4^aA^	80.5^aB^	85.8^aAB^	-	-	-
	PP	61.1^cC^	70.4^bB^	78.0^bA^	-	-	-

T, treatment effect; S, storage temperature effect; T×S, interaction effect between treatment and storage temperature; DM, dry matter; CK, control; LP, *Lactobacillus plantarum* KR107070; PP, *Pediococcus pentosaceus* 17604; CP, crude protein; WSC, water soluble carbohydrates; EE, ether extract.

CP, WSC, and CF were expressed on DM% and nitrate was shown as mg/kg (DM).

Means within columns (^a,b,c,d^) or rows (^A,B,C,D^) with different letters are significantly different (p<0.05).

**Table 7:** Mycotoxin content of corn silage treated with lactic acid bacteria and storage temperature

Item	Treatment	Storage temperature (°C)			p-value		
	
		20	28	37	T	S	T×S
NIV	CK	39.5^bC^	55.8^bB^	137.1^aA^	<0.0001	<0.0001	<0.0001
	LP	39.1^bB^	91.7^aA^	87.4^bA^	-	-	-
	PP	59.6^aB^	50.8^cC^	68.8^cA^	-	-	-
DON	CK	56.4^bA^	42.5^bB^	38.3^cB^	< 0.0001	< 0.0001	< 0.0001
	LP	76.2^bC^	126^aB^	179^aA^	-	-	-
	PP	261^aA^	44.1^bC^	66.2^bB^	-	-	-
3-ADON	CK	38.9	36.8	37.6	0.1870	0.1871	0.1022
	LP	37.0	37.0	37.5	-	-	-
	PP	37.1	37.2	37.3	-	-	-
AFB_1_	CK	24.5	23.9	24.2	0.3383	0.4968	0.7451
	LP	23.6	23.6	24.3	-	-	-
	PP	24.0	23.4	23.7	-	-	-
HT-2	CK	13.4	13.3	13.6	0.7065	0.7426	0.6698
	LP	13.4	13.8	13.0	-	-	-
	PP	13.8	13.6	13.5	-	-	-
T-2	CK	21.9	22.3	22.2	0.7535	0.0121	0.4168
	LP	21.9	22.7	22.0	-	-	-
	PP	22.2	22.4	22.1	-	-	-
OTA	CK	34.7^B^	34.9^bB^	51.6^aA^	0.0001	< 0.0001	< 0.0001
	LP	36.2^B^	36.2^bB^	39.2^cA^	-	-	-
	PP	36.5^C^	40.7^aB^	44.1^bA^	-	-	-
ZEA	CK	27.5^b^	30.4^b^	29.6	< 0.0001	< 0.0001	< 0.0001
	LP	28.0^bAB^	27.4^bB^	29.4^A^	-	-	-
	PP	37.0^aB^	62.0^aA^	30.6^B^	-	-	-

Mycotoxins were shown on dry matter basis (ng/g).

T, treatment effect; S, storage temperature effect; T×S, interaction effect between treatment and storage temperature; NIV, nivalenol; CK, control; LP, *Lactobacillus plantarum* KR107070; PP, *Pediococcus pentosaceus* 17604; DON, deoxynivalenol; 3-ADON, 3-acetyl-deoxynivalneol; AFB_1_, aflatoxin B_1_; HT-2, HT-2 toxin; T-2, T-2 toxin; OTA, ochratoxin A; ZEA, zearalenone.

Means within columns (^a,b,c,d^) or rows (^A,B,C,D^) with different letters are significantly different (p<0.05).
